# Gestational Diabetes Mellitus: The Dual Risk of Small and Large for Gestational Age: A Narrative Review

**DOI:** 10.3390/medsci13030144

**Published:** 2025-08-19

**Authors:** Andreea Fotă, Aida Petca

**Affiliations:** 1 Department of Obstetrics and Gynecology, “Carol Davila” University of Medicine and Pharmacy, 8 Eroii Sanitari Blvd., 050474 Bucharest, Romania; aida.petca@umfcd.ro; 2 Department of Obstetrics and Gynecology, Elias University Emergency Hospital, 17 Mărăști Blvd., 050474 Bucharest, Romania

**Keywords:** gestational diabetes mellitus, fetal growth, small for gestational age, large for gestational age, low birth weight, macrosomia, intrauterine growth restriction

## Abstract

**Background/Objectives**: Gestational diabetes mellitus (GDM) complicates approximately 14% of pregnancies worldwide, its prevalence rising with increasing maternal age and obesity. While maternal hyperglycemia is traditionally associated with fetal overgrowth and large-for-gestational-age (LGA) neonates, emerging evidence indicates that GDM may also contribute to small-for-gestational-age (SGA) outcomes. **Methods**: A comprehensive literature search was conducted using multiple databases, including PubMed, Web of Science, and ScienceDirect, to identify studies related to gestational diabetes mellitus, fetal growth outcomes such as small for gestational age and large for gestational age, and associated pathophysiological mechanisms. **Results**: This narrative review explores the mechanisms by which GDM influences fetal growth, emphasizing the dual risk of excessive and restricted intrauterine growth. Fetal macrosomia typically results from chronic maternal hyperglycemia, leading to increased transplacental glucose delivery and fetal hyperinsulinemia. In contrast, SGA outcomes are a consequence of vascular and endothelial dysfunction, placental insufficiency, or excessively restrictive glycemic control that limit the availability of nutrients. Both extremes of fetal growth carry a myriad of significant perinatal and long-term metabolic risks. **Conclusions**: Understanding the diverse pathways through which GDM affects fetal growth is essential for developing individualized clinical strategies.

## 1. Introduction

Gestational diabetes mellitus (GDM) is one of the most prevalent gestational complications, characterized by high glycemic levels with onset or first recognition during pregnancy. GDM is divided into classes: the primarily diet-controlled GDM and GDM requiring pharmacologic treatment. According to the International Diabetes Federation, gestational diabetes is estimated to affect around 14% of pregnancies worldwide [1]. In Europe, GDM has a prevalence of almost 11%, with the highest rates noted in Eastern European countries: 31.5% [2]. The incidence of GDM is expected to increase, especially due to the rising prevalence of obesity among women of reproductive age, as well as an aging population, urbanization, sedentarism, and stressful lifestyles, particularly in middle- and upper-income countries [3].

Apart from the increased direct costs of managing GDM, including screening, monitoring, pharmacological and dietary therapies, hospitalization, and neonatal care costs, the indirect and intangible costs are insufficiently discussed. These include reduced productivity at work, absenteeism, poor emotional health, physical pain, and decreased quality of life [4]. Due to its significant role in increasing the risk of developing type 2 diabetes, obesity, cardiovascular diseases, and metabolic disease, for both the mother and her offspring [5], GDM is a crucial public health concern. Diabetes mellitus is diagnosed in approximately 10% of women soon after delivery, while 20–60% of the rest appear to develop diabetes mellitus within 5–10 years after pregnancies associated with GDM, in the absence of specific therapeutic interventions [5].

Generally, GDM increases the risk of fetal overgrowth, with fetuses being evaluated as being large for gestational age (LGA) or macrosomatic. Recent evidence shows that up to 7% of infants born to women with gestational diabetes are small for gestational age (SGA), particularly when maternal glucose is overtreated or when additional risk factors co-exist, such as low body mass index (BMI), preeclampsia, and vascular insufficiency. The pregnancies that are associated with SGA fetuses face elevated risks of preterm birth and stillbirth, as well as neonatal hypoglycemia, seizures, respiratory distress, and maternal postpartum hemorrhage [6].

Fetal macrosomia is defined as a birth weight of ≥4000 g. The underlying mechanism of macrosomia is still often referred to as “Pedersen’s Hypothesis” [7]. Maternal hyperglycemia leads to increased glucose transfer through the placenta to the fetus. This stimulates the fetal pancreas towards hyperinsulinemia, which has an anabolic effect and leads to LGA or macrosomia. These are associated with a higher occurrence of birth complications, including, but not limited to, shoulder dystocia, neonatal hypoglycemia, and abnormal lung maturation [8].

Small for gestational age is defined as birth weight < 2700 g or below the 10th percentile for gestational age and sex. Intrauterine growth restriction (IUGR) is, however, linked to mechanisms referring to maternal microangiopathy, altered lipid and amino acid parameters, and elevated inflammatory markers. Several inflammation markers, associated with oxidative stress and endothelial dysfunction, have been linked to IUGR, including C-reactive protein, interleukin-6, tumor necrosis factor-alpha, and adiponectin [9,10].

The majority of the medical focus has been on preventing LGA fetuses during GDM management; however, the literature underscores the need to also identify and prevent SGA. Studying both extremes of fetal growth in GDM leads to an improved understanding of maternal and fetal health, including the underlying mechanisms, risk factors, and management.

Personalized care, earlier screening, and balancing glycemic control to mitigate risks of both fetal overgrowth and undergrowth should be considered the primary strategies in GDM management.

## 2. Materials and Methods

**Figure 1:**
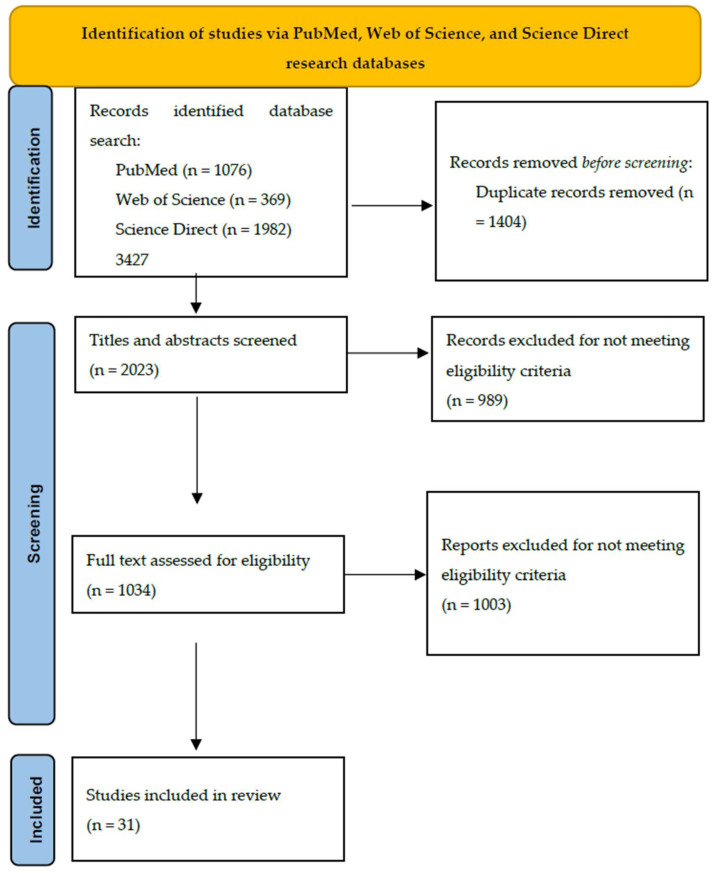
Identification of studies via PubMed, Web of Science, and ScienceDirect research databases.

This paper is a narrative review of the specialty literature published in English. A comprehensive literature search was conducted using multiple databases, including PubMed, Web of Science, and ScienceDirect, to identify studies related to gestational diabetes mellitus, fetal growth outcomes such as small for gestational age and large for gestational age, and associated pathophysiological mechanisms and risk factors (Table 1). Studies published from inception to June 2025 were included (last search date: 30 June 2025). A detailed process is illustrated in Figure 1.

The search strategy included utilizing combinations of specific keywords occurring in either the titles or the abstract of the articles, including “gestational diabetes mellitus”, “GDM”, “small for gestational age”, “intrauterine growth restriction”, “low birth weight”, “macrosomia”, and “large for gestational age”.

Inclusion criteria encompassed clinical studies (randomized controlled trials, observational studies, and case series). We excluded systematic reviews and meta-analyses from our search, as well as studies discussing the outcomes of pharmacological interventions. Duplicates, articles lacking methodological clarity or data, animal studies, articles published in languages other than English, as well as systematic reviews and meta-analyses were also excluded. Because our aim in this narrative review was to provide an original perspective based on clinical trials, we limited our analysis primarily to such studies, incorporating systematic reviews and meta-analyses only in the Discussion section to contextualize our findings within the existing literature.

## 3. Results

A total of 3427 studies were evaluated. After the exclusion of 3396 articles, a total of 31 studies that evaluated ethnicity, age, lipid parameters, pre-pregnancy BMI, maternal diet, serum levels of ferritin, vitamin D, vitamin E, free fatty acids, and maternal hemodynamic and vascular changes were included. In order to ease readability, the results were further grouped based on the analyzed risk factors. Table 2 presents all included studies, with emphasis on primary outcomes—SGA/intrauterine growth restriction/low birth weight and LGA/macrosomia—highlighting the dual risk of fetal growth abnormalities by enabling direct comparison of these outcomes across studies.

### 3.1. Ethnicity

Several studies assessed the frequency of adverse pregnancy outcomes among GDM patients based on ethnicity. Particularly, Africans, Asians, Pacific Islanders, and Latinos are considered to have the highest risk of GDM.

In an extensive study published by Venkatesh et al. [11], in which 1,560,722 pregnant women were included, 1% were American Indian, 13% were Asian/Pacific Islander, 12% were Black, 27% were Hispanic/Latina, and 48% were White. From the beginning of the study, in 2014, until 2020, a statistically significant decrease in the overall incidence was reported in LGA and macrosomia percentages (−2.3% and −4.7%, respectively); there were no notable changes in the frequency of SGA. Black individuals had a higher risk of SGA and American Indian individuals, of LGA and macrosomia; Hispanic/Latina and Asian/Pacific Islanders individuals had a significantly increased risk of SGA. Differences in adverse outcomes by race and ethnicity persisted throughout the duration of the study.

Filardi et al. [12] published a study on 399 patients with GDM, of whom 76 were of high-risk (HR) ethnicity (71.1% Asian, 15.8% African, and 13.1% Hispanic). The low-risk (LR) population consisted of Caucasians. Fetal growth abnormalities were evaluated in 28 high-risk pregnancies and 83 low-risk pregnancies, respectively. Statistically significant differences were reported: LGA was identified in 21.4% of the HR compared to 7.2% of the LR population; SGA was noted in 14.3% of the HR and 4.8% of the LR populations, even after adjustment for confounding factors.

Ten racial/ethnic groups, comprising Hispanic, non-Hispanic white (NHW), non-Hispanic black (NHB), Filipino, Chinese, Asian Indian, Vietnamese, Korean, Japanese, and Pacific Islander, with infants born to mothers with GDM, were assessed in a study published by Xiang et al. [13] in 2015. The study population included a retrospective cohort of 342,496 women, out of which 29,544 had GDM. Of those, 57.4% were Hispanic, 20.1% were NHW, 7.2% NHB, 7.2% Filipino, 2.4% Chinese, 2.2% Asian Indian, 2% Vietnamese, 0.6% Korean, 0.4% Japanese, and 0.5% Pacific Islander. LGA risks ranged from 9.6 to 17.2%, and SGA risks ranged from 5.6 to 11.3%. The highest LGA risk was reported in the NHB population (17.2%), followed by PI (16.2%), Hispanic (14.5%), NHW (13.1%), Asian Indian (12.8%), and Filipino (11.6%). The SGA risks were 11.3% in the Filipino population, 10.4% in the Asian Indian population, 9.5% in the NHW population, 9.4% in the Vietnamese population, 8.9% in the Hispanic population, 7.5% in the NHB population, and 5.6% in the PI population. Chinese, Korean, and Japanese women registered the lowest risks for both SGA and LGA. After adjustments, only NHB had a significantly increased risk of LGA compared to NHW, and none of the other racial/ethnic groups had a statistically significant risk of SGA compared to NHW.

Nielsen et al. [14] investigated singleton deliveries from GDM pregnancies among migrants in Denmark. The women were from Turkey, the Former Soviet Union, Former Yugoslavia, Iraq, Somalia, Poland, Lebanon, Pakistan, Afghanistan, Germany, China, Morocco, Sri Lanka, and India. Out of the cohort consisting of 710,413 deliveries, 2.6% were associated with GDM, and 14.4% were among immigrants. The presence of GDM doubled the odds of LGA among women from all countries; this was not statistically significant among women from India or China. Heightened risks were noted in women from Sri Lanka (odds ratio (OR): 4.20). Among Danish women, SGA proved to have lower odds (OR 0.90). Forty to fifty percent reduced odds of SGA were noted in populations from countries such as the Former Yugoslavia, Turkey, Somalia, Afghanistan, Sri Lanka, Pakistan, and other non-Western countries. The odds for LGA were significantly lower among women from India, Lebanon, Pakistan, Iraq, Turkey, and Somalia, compared to Danish women. Except Turkey, women from these countries had higher odds of SGA, in comparison with Danish women.

### 3.2. Maternal Age

Zhang et al. [15] published a study on 24,551 Chinese pregnant women with GDM, divided into five groups based on age (20–24, 25–29, 30–34, 35–39, and 40–44 years old). The prevalence of adverse outcomes was the lowest in the 25–29 years old group, and therefore, it was considered the reference group. Compared to the aforementioned population, women aged 35–44 had a significantly higher risk of LGA and macrosomia; women aged 40–44 had a higher risk of small for gestational age. Pregnant women aged 20–24 years had the lowest rate of macrosomia (9.7%), and those aged 35–59 had the highest rate (13.2%). The lowest rate of SGA was recorded in the 30–34 group (15.4%), and the highest rate was noted in the 40–44 group, 19.7%. The 20–24 group had the lowest rate of LGA (14.9%), while the 35–39 and 40–44 groups had the highest rates, 21.1% and 21.2%, respectively.

Another study, published by Lu et al. [16], evaluated the risks of adverse infant outcomes in women aged 45 and over from the United States. A total of 52,544 women were included. GDM was associated with a significantly reduced risk of low birth weight. No associations were found between GDM and SGA in women over 45 years old.

### 3.3. Maternal Lipid Parameters

A physiological increase in blood lipid levels occurs during pregnancy, with gradual increases culminating in a peak in the late stages. The highest increase is reported regarding triglyceride levels, whose role as a primary energy source is accompanied by protein synthesis, a decrease in fat breakdown, and facilitation of nutrient accumulation.

Five studies discussed the impact of lipid profile changes on the development of GDM.

Krstevka et al. [17] analyzed 200 singleton pregnancies with GDM and 43 with type 2 diabetes mellitus in the third trimester. Maternal serum triglycerides (TG), low-density lipoprotein (LDL) cholesterol, and total cholesterol concentrations were independent predictors of LGA. High-density lipoprotein (HDL) cholesterol and HbA1c were not directly correlated with LGA.

However, another cohort study by Pereira et al. [18] included 659 women, out of which 56 had LGA, 56 had SGA, and the rest had appropriate-for-gestational-age (AGA) neonates. Lower levels of HDL cholesterol were noted for women with LGA infants compared to the other two groups, as well as for women with AGA infants compared to women with SGA infants. Unlike the previous study, Pereira did not observe associations between TG, total cholesterol, or LDL cholesterol levels and the occurrence of LGA.

Simeonova et al. [19] analyzed 200 GDM pregnancies, out of which 50 delivered LGA newborns, and 15 delivered SGA infants. In the LGA and SGA groups, compared to the AGA group, maternal TG and HbA1c levels in the second trimester were higher. Moreover, in the LGA group, compared to the AGA group, HDL cholesterol was significantly lower.

Peng et al. [20] published a retrospective study, including 10,490 singleton pregnancies, out of which 2351 were associated with GDM and 8139 were not. TG levels were directly correlated with birth weight in both groups, whereas other parameters (total cholesterol, HDL cholesterol, and LDL cholesterol) were mildly negatively correlated with birth weight. In the GDM group, each 1 mmol/L TG increase correlated with a 28.4 g increase in birth weight, whereas the same increases of either TC, HDL cholesterol, or LDL cholesterol were associated with birth weight decreases. In the GDM group, high TG levels (above the 90th percentile) were associated with increased birth weight compared to low TG levels (below the 10th percentile); high TG, HDL cholesterol, and LDL cholesterol levels were associated with decreased birth weight. Peng’s study showed no association between lipid levels in pregnancies with GDM and the risk of SGA.

### 3.4. Pre-Pregnancy BMI

Ten studies discussed the role of pre-pregnancy BMI in the development of fetal growth abnormalities.

A retrospective observational cohort study of 308 women, published by Drever et al. [21], reported on potential predictors of SGA infant delivery. Metabolic risk factors associated with SGA included lower pBMI and lower fasting blood glucose level. Ultrasound-guided high SGA risk based on either low abdominal circumference, low estimated fetal weight, or both was also considered a significant predictor for SGA. In spite of being at a high risk for SGA, the majority of the population did not receive additional therapeutic management.

Yang et al. [22] explored the mediating role of gestational diabetes in the relationships between pre-pregnancy BMI (pBMI) and maternal and infant complications. The study, encompassing 6174 pregnant women, showed that the patients with obesity had a higher risk for macrosomia and large for gestational age; 4.61% and 5.02% of the associations were mediated by gestational diabetes mellitus. Underweight women had a higher risk for low birth weight and small for gestational age.

Similarly, in Kondracki’s study [23], GDM was evaluated as a mediator in causal mediation analyses assessing how pBMI influences the risk of LGA births. Out of 3,229,783 singleton term births, 6.4% of women had GDM, and 3.6% were LGA singleton term births. The total effect estimated that the pre-gestational overweight/obesity group had the highest rates of LGA births at term. Natural direct effect estimates were higher than natural indirect effect estimates for patients with higher-than-normal BMI statuses. The results demonstrated that the fraction of the total effect of increased BMI on LGA births that operates through GDM was highest for early-term deliveries, reaching approximately 16%.

A retrospective cohort study involving 13,467 women, published in 2022 by Chen Xu [24], demonstrated an association between pre-gestational overweight and obesity and a reduced risk of small for gestational age. Obesity alone was associated with an increased risk of macrosomia and large for gestational age, as well as a decreased risk of low-birth-weight infants.

Liao’s study [25], encompassing 791 pregnant women with GDM, explored the association between maternal pBMI and neonatal birth weight. The percentage of both LGA and SGA neonates was directly associated with the increase in pBMI. Moreover, when pBMI was lower than 27.78 kg/m^2^, neonatal birth weight significantly increased alongside the increase in pBMI. When maternal pBMI was higher than that, neonatal birth weight decreased as pBMI increased.

A nationwide study conducted in Japan by Saito et al. [26] and published in 2022 collected data from the national birth cohort. Out of 85,228 patients, 2.6% (2216) developed GDM. The highest percentage of SGA was 12%, noted in the underweight GDM group. The odds ratio (OR) of SGA was elevated in the overweight/obese GDM group compared to non-GDM patients with overweight/obesity. The odds ratio of LGA was also significantly higher in the overweight/obese GDM group, compared to non-GDM patients with normal weight, overweight, or obesity.

Hu et al. [27] evaluated 17,260 singleton pregnancies, out of which 1355 developed GDM. The study found no effects of maternal pBMI on the risk of SGA; on the other hand, GDM significantly mediated the association between overweight/obese pBMI and LGA.

Mothers with GDM and overweight/obesity were at higher risk of LGA than those who had GDM or overweight/obesity, as noted by Chen et al. [28].

Another article, published in 2022 by Song [29], showed an incidence rate of GDM of 12.3% within a population of 15,065. SGA occurred in 6.4% of the GDM participants and LGA in 11.9% compared to the non-GDM population (5% and 9.6%, respectively). Pregnant women with overweight or obesity before pregnancy (preBMI ≥ 24 kg/m^2^) had a higher risk of delivering large-for-gestational-age infants and a lower risk of small-for-gestational-age infants compared to those with normal preBMI. Similarly, women with gestational diabetes mellitus also showed an increased risk of LGA and a higher likelihood of SGA.

In Pereira’s study [18], mothers with LGA infants had a statistically significant greater pre-pregnancy BMI than mothers who had given birth to AGA and SGA infants, respectively (32.1 vs. 29.8 vs. 28.7).

### 3.5. Maternal Diet

Few studies have aimed to determine whether a detailed lifestyle program, including nutritional advice and the intake of specific micro- and macronutrients, influences the outcomes of GDM.

In one randomized controlled trial conducted by Bruno et al. [30], 191 women with a pre-pregnancy BMI of over 25 kg/m^2^ were randomized into two groups: the first group received recommendations on diet and physical activity, whereas the second group was prescribed a personalized dietary plan. The occurrence of GDM was significantly lower in the second group (18.8% vs. 37.1%). The incidence of LGA neonates and macrosomia was significantly lower in the second group (one vs. seven for LGA; two vs. seven for macrosomia); the incidence of SGA was similar between the groups.

Another prospective cohort study by Apostolopoulou et al. [31] included 90 patients with GDM and assessed the risk of SGA neonates, based on the intake of micro- and macronutrients. The participants were surveyed about their dietary habits six months prior to pregnancy and from the onset of pregnancy until the diagnosis of GDM at 24–28 weeks of pregnancy via an oral glucose tolerance test (OGTT). Notably, water intake was associated with a decrease in SGA risk. From the onset of pregnancy, carbohydrate and fiber intakes were associated with a 5% and 21% lower risk of SGA, respectively, while fat intake increased the risk by 10%. Added sugar intake lowered the risk by 4%. Magnesium and copper intakes during the second period were also statistically significant in relation to SGA. Regarding certain food items, fresh salad and sunflower oil were associated with SGA during the second period, while legume intake was significant during the first period. The association was increased when frying was the preferred cooking method during the second period.

A similarly conceived study by Siargkas et al. [32] including 117 GDM patients assessed macronutrient intake six months before pregnancy and until the diagnosis of GDM via OGTTs. Among women with a normal pre-pregnancy BMI, higher intakes of dietary fiber and vegetable protein before pregnancy were significantly associated with an increased risk of delivering an LGA infant. The increased risk associated with vegetable protein intake was maintained during early pregnancy. In contrast, among women with overweight or obesity, no significant associations were found between pre-pregnancy dietary factors and LGA risk. However, during early pregnancy, a higher proportion of total carbohydrate intake was associated with an increased risk of LGA, while maintaining saturated fat intake “as low as possible” was linked to a reduced risk. Higher vegetable protein intake during early pregnancy was also associated with an increased risk of LGA.

High cholesterol intake during the second trimester led to higher risks of macrosomia in pregnancies with GDM but lower risks of SGA compared to those with low cholesterol intake according to a prospective cohort study on 349 women published by Xie et al. [33] in 2024. In the low-cholesterol intake group, comprising 175 women, the average cholesterol intake was 489.31 ± 131.56 mg/day, whereas in the high-cholesterol intake group (174 women), it was 820.85 ± 162.36 mg/day. In the third trimester, high cholesterol intake was associated with lower risks of macrosomia and LGA compared to those with low cholesterol intake.

A randomized controlled trial published in 2022 by Messika et al. [34] assessed whether sleep hygiene and chrononutritional interventions could improve maternal glycemic control in a population of 103 women with GDM. While the intervention group (33 patients) benefitted from a significantly improved glycemic control due to the reduced carbohydrate intake in the evening, the program had no effect on the proportion of LGA neonates.

Cheng et al. [35] evaluated the duration of folic acid supplementation and its impact on the development of GDM and other associated birth outcomes. Folic supplementation of over 3 months before pregnancy is associated with an increased risk of macrosomia. However, during pregnancy, over 3 months of supplementation might decrease the effect of GDM on macrosomia. The mechanism may be linked to an increase in maternal and cord blood folate levels, leading to lower homocysteine concentrations and decreased birth weight. Moreover, maternal plasmatic folic acid levels influence deoxyribonucleic acid (DNA) methylation, and folic acid provides methyl groups for biochemical reactions. Neonatal DNA methylation is related to birth weight [42,43].

### 3.6. Biochemical Markers and Vascular Adaptation

A study published in 2010 by Soubasi et al. [36] aimed to determine whether serum ferritin levels were linked to GDM and IUGR. High maternal ferritin levels were significantly associated with increased risks of GDM and IUGR.

The effect of vitamin D deficiency (<20 ng/mL) in pregnancies with GDM has been evaluated by Weinert et al. [37]. Out of two hundred thirty women with GDM, ninety-eight had vitamin D deficiency. The incidence of SGA was higher in the group of vitamin D deficiency (17.3% vs. 5.9%). Moreover, maternal vitamin D deficiency was associated with higher incidences of neonatal hospitalization in intensive care units and neonatal hypoglycemia.

Vitamin E has an established role in mitigating oxidative stress. In a retrospective study involving 19,647 women published by Zhou et al. [38], maternal serum vitamin E levels were assessed in each trimester. Women who developed GDM showed consistently higher vitamin E levels throughout pregnancy compared to those without GDM. Additionally, pregnancies resulting in LGA infants were characterized by higher maternal vitamin E levels in the first and second trimesters compared to those with non-LGA infants. After adjusting for potential confounding factors, excessive vitamin E levels during the second trimester remained an independent risk factor for both GDM and LGA. Overall, maternal vitamin E concentrations in the first and second trimesters demonstrated a positive association with the development of GDM and the incidence of LGA.

Hyperuricemia in early pregnancy is associated with several adverse outcomes. A total of 18,250 pregnant patients, out of which 2896 (15%) with high blood uric acid levels, were included in an analysis by Pang et al. [39]. The mean maternal uricemia was 0.22 ± 0.05 mmol/L. Hyperuricemia was associated with a 39.4% higher risk of GDM but not with macrosomia, SGA, and LGA.

Kim et al. [40] investigated the association between free fatty acid (FFA) levels and LGA newborns in women with GDM. Plasma lipid profiles were measured, including fasting and 2 h postprandial FFA levels, between 24 and 32 weeks of gestation, after eating a standard mixed meal (480 kcal, 60% carbohydrate, 20% protein, and 20% fat). Out of 710 enrolled pregnant women, 59% had LGA newborns. Postprandial FFA levels were higher in women with LGA newborns compared with women with non-LGA newborns (416.7 μEq/L vs. 352.5 μEq/L). Fasting FFA showed no statistically significant differences between the two groups. There were no reported differences between maternal TG levels of those who delivered LGA compared to those who delivered non-LGA newborns.

Chatzakis et al. [41] published a study in 2025 that aimed to evaluate maternal hemodynamic and vascular changes in pregnancies with abnormal fetal growth. Eleven percent of 11,132 women developed GDM. In the aforementioned population, three parameters showed a positive correlation with the development of SGA: total peripheral resistance, ophthalmic artery peak systolic velocity, and uterine artery pulsatility index. The SGA groups of both non-GDM and GDM pregnancies had the highest uterine artery pulsatility index percentiles, carotid–femoral pulse wave velocity, and ophthalmic artery peak systolic velocity ratios. Compared to AGA groups, cardiac output was lower in the SGA groups.

## 4. Discussion

Women diagnosed with GDM face a significantly higher risk of adverse obstetric and neonatal outcomes compared to those with normoglycemic pregnancies. GDM is particularly associated with increased incidences of fetal macrosomia, shoulder dystocia, birth trauma, and many other adverse outcomes [44,45,46]. Multiple studies have proved that the risk of fetal anomalies is closely associated with glycosylated hemoglobin levels during pregnancy, most commonly affecting the cardiovascular or musculoskeletal systems [47,48,49].

In 2013, the World Health Organization (WHO) adopted the International Association of Diabetes and Pregnancy Study Groups (IADPSG) criteria for the diagnosis of GDM. These criteria are based on a 75 g oral glucose tolerance test. The diagnostic cut-off values are defined as fasting glucose ≥ 5.1 mmol/L (92 mg/dL), 1 h ≥ 10.0 mmol/L (180 mg/dL), and/or 2 h ≥ 8.5 mmol/L (153 mg/dL) [50].

The implementation of these revised criteria, characterized by lower fasting thresholds, the inclusion of a 1 h measurement, diagnosis based on a single abnormal value, and the use of a one-step diagnostic approach without prior screening, has led to a reported 2–11-fold increase in GDM prevalence compared to previous diagnostic standards [51]. This rise parallels the global increase in obesity and type 2 diabetes, particularly among younger adults [52]. Consequently, the growing number of GDM diagnoses has raised concerns regarding the burden on healthcare systems, potential impacts on maternal and neonatal health outcomes, quality of life, and healthcare costs [4,53].

The latest data estimate an international prevalence of GDM of up to 14.7%, according to a study by Saeedi et al. [46], which includes an exhaustive meta-population analysis based on IADPSG guideline criteria.

Discordant fetal growth in the context of gestational diabetes has an intricate pathophysiology. Gestational diabetes impacts fetal development, and while macrosomia is the most known outcome, this pathology can also lead to intrauterine growth restriction. Significant structural alterations of the placenta are involved, which compromise its function. Placentas from GDM pregnancies are typically enlarged and exhibit increased surface area, thickness, and weight, with histopathological changes such as cytotrophoblastic hyperplasia, fibrinoid deposition, immature and edematous villi, and thickening of the syncitiotrophoblast base membrane [54]. These structural modifications increase the distance between the maternal intervillous space and the fetal vasculature, impairing nutrient and oxygen exchange and increasing the risk for both fetal macrosomia and growth restriction [55].

Hyperglycemia in the placenta increases reactive oxygen species (ROS) levels and lipid peroxidation, leading to mitochondrial dysfunction in trophoblasts. Excessive ROS can damage key cellular components—proteins, lipids and DNA—impairing cellular function and physiological processes. Oxidative damage is a factor in inflammatory responses, compromising the integrity of cellular structures. In the context of diabetes, oxidative stress contributes to the pathogenesis of the disease and exacerbates its progression. An imbalance in the production of constricting factors vs. relaxing factors reflects endothelial dysfunction. Oxidative stress is a critical driver of both the initiation and progression of vascular dysfunction, as well as promotion of systemic inflammation. Released mediators, such as ROS, metalloproteinases, cytokines, and chemokines, lead to vascular injury, vasoconstriction, and structural remodeling of the vessel wall [56]. Vascular dysfunction within the placenta increases the risk of IUGR. GDM is associated with both maternal and fetal vascular malperfusion, marked by reduced spiral artery remodeling, increased syncytial knots, delayed villous maturation, and perivillous fibrin deposits. These vascular lesions inhibit perfusion and nutrient delivery, exacerbating hypoxia and placental insufficiency [57].

On the other side of the spectrum, maternal hyperglycemia leads to an increased transport of glucose and subsequent anabolic state that leads to fetal excessive growth. Adiponectin has also been cited as a factor, impairing placental amino acid transport and therefore restricting fetal growth; however, in GDM, adiponectin production is diminished through gene methylation. Additionally, microarray analyses comparing placentas from patients with and without GDM have identified microRNA alterations in GDM that may increase epidermal growth factor receptor signaling, leading to an enhanced fetal growth [58].

During pregnancy, physiological changes in maternal lipid parameters are characterized by progressive increases in serum TG, total cholesterol, and lipoproteins, peaking in the third trimester to meet fetal energy demands. These changes, while expected, may be exaggerated or pathologically altered in pregnancies complicated by GDM. These lipid abnormalities reflect increased insulin resistance and altered lipid clearance [59]. Furthermore, increased maternal serum FFAs can cross the placenta and contribute to excess fetal growth [60]. In the study that we included in our review [40], postprandial FFA levels were significantly associated with LGA infants; monitoring postprandial FFA may provide important insight when managing patients at risk.

Elevated maternal TG and FFA levels, especially in the second half of pregnancy, are strongly associated with an increased risk of delivering LGA newborns in GDM and diabetic pregnancies, independent of glycemic control, pre-pregnancy BMI, or gestational weight gain [61,62]. While HDL cholesterol tends to be inversely related to fetal overgrowth, its role remains less consistent across studies. The contribution of other cholesterol fractions, such as LDL and total cholesterol, appears to vary depending on study design and population characteristics.

The relationship between dyslipidemia and small for gestational age is less clear but still notable. Systematic reviews yield inconsistent results: while some studies report no significant associations, others suggest that maternal dyslipidemia may contribute to SGA, perhaps via impaired placental lipid transport or altered nutrient availability [63,64]. A study evaluating 150 pregnancies with GDM that evaluated maternal serum and cord blood levels of lipid parameters, cord blood TG, and cholesterol levels correlated negatively with birth weight. Moreover, cord blood TG levels were higher in SGA infants compared with both AGA and LGA neonates, possibly reflecting reduced lipoprotein lipase activity and impaired fetal lipid utilization. Insulin, insulin-to-glucose ratios, and FFA levels in cord blood were the highest in LGA infants [62].

A recent systematic review and meta-analysis focused on Chinese pregnant women with GDM. It reported that the participants with overweight or obesity had significantly higher risks for LGA and lower risk of SGA compared with participants of normal weight. Women who were underweight had a higher risk of SGA and reduced LGA and macrosomia risks [65]. Maternal obesity plays a significant role in the risk of fetal overgrowth. In our review, pBMI belonging to the overweight and obese ranges was consistently associated with increased risks of LGA and macrosomia. Conversely, underweight pBMI increased the risks of SGA and LBW. Notably, Liao’s [25] analysis found a non-linear association, where neonatal birth weight increased with pBMI up to a threshold (27.8 kg/m^2^), beyond which further increases in pBMI were associated with a decrease in birth weight. This suggests a complex relationship, potentially influenced by metabolic, inflammatory, or vascular factors associated with extreme maternal obesity. GDM partially mediates these effects, particularly in the setting of elevated pBMI, but does not entirely explain the relationship, indicating the need for a broader clinical focus on maternal weight optimization before conception.

A 2019 systematic review and meta-analysis published by Chia et al. [66] included 36 studies discussing dietary patterns of 167,507 participants and revealed that “healthy” diets rich in fruits, vegetables, whole grains, low-fat dairy, and lean proteins were associated with a weak trend toward lower SGA risk and slightly higher birth weights (mean difference: 67 g), whereas “unhealthy” patterns (refined grains, processed meats, saturated fats, sugars) correlated with reduced birth weight (−40 g) but showed no consistent associations with SGA or LGA risks. The studies included in our review showed that lifestyle modification and targeted nutritional interventions can reduce the risk and severity of GDM fetal growth abnormalities. Micro- and macronutrient intake during preconception and early pregnancy has successfully modified fetal growth patterns in GDM pregnancies. A reduced SGA risk was observed in patients with increased intake of carbohydrate and fiber, whereas higher fat intake increased the risk of SGA. Paradoxically, another study by Siargkas et al. [32] identified that increased preconception intake of fiber and vegetable protein was linked to a higher LGA risk among women with normal BMI. During early pregnancy, higher carbohydrate intake and lower saturated fat intake correlated with increased and decreased LGA risk, respectively.

Similarly, magnesium, copper, and specific food choices can influence the SGA risk, further emphasizing the relationship between maternal diet and fetal development. Dietary behaviors such as frying were associated with an increased risk of SGA, highlighting not just what is consumed but also how it is prepared and consumed. Cholesterol intake is also an influential factor in fetal growth. Higher intake during the second trimester was linked to a higher risk of macrosomia but a lower risk of SGA. In the third trimester, higher cholesterol intake was associated with lower risks for both macrosomia and LGA.

Reduced evening carbohydrate intake improved glycemic control in GDM patients, but it did not impact the incidence of LGA. While circadian eating patterns and recommendations can benefit the maternal outcomes, their impact on fetal outcomes might require longer-term follow-up.

Folic acid supplementation, typically 400 micrograms daily, starting 3 months preconceptionally, significantly reduces the risk of neural tube defects (NTDs) such as spina bifida and anencephaly [67]. In a systematic review of 31 trials, involving 17,771 women, supplementation was associated with a mean birth weight increase (~136 g) and a substantial reduction in megaloblastic anemia, although no statistically significant effects were observed on preterm birth or neonatal death rates [68]. According to the study that we have selected, folic acid supplementation before and during pregnancy seems to have a dual effect. Prolonged preconceptional folic acid supplementation, over 3 months, was associated with an increase in the risk of macrosomia, whereas prolonged supplementation during pregnancy appeared to lower the effects of GDM on birth weight.

Recent evidence underscores the influence of maternal biochemical and vascular factors on GDM and associated fetal growth abnormalities. Maternal iron status may contribute to oxidative stress or inflammation, as reported by one study: elevated maternal serum ferritin levels have been independently linked to increased risks of both GDM and IUGR. Micronutrient reduced and elevated levels also seem to impact pregnancy outcomes. A 2019 umbrella review by Iqbal and Ekmekcioglu [69] summarizing 16 meta-analyses found that iron supplementation can reduce maternal anemia and low-birth-weight risk, but excessive iron levels are associated with increased GDM incidence and can influence birth size outcomes.

Vitamin D deficiency is highly prevalent among fertile women: a systematic review conducted by Lucchetta et al. [70] that included 31 studies found that 35% were deficient, and 42% had insufficient vitamin D levels. Vitamin D deficiency was significantly associated with higher rates of SGA infants, increased neonatal intensive care unit admissions, and a higher incidence of neonatal hypoglycemia.

Conversely, vitamin E, while usually considered protective against oxidative stress, was found to have paradoxically heightened levels in women with GDM. Elevated vitamin E levels in the first and second trimesters were also associated with an increased risk of delivering LGA infants, suggesting a potential disruption in the metabolic processes during pregnancy. Elevated maternal vitamin E levels result from dietary overconsumption of tocopherol-rich foods, large-dose supplementation (over 400 international units/day), or concomitant antioxidant therapies during pregnancy [71].

Hyperuricemia may be an early metabolic marker of GDM risk, as it was associated with a 39.4% increased risk for developing GDM. In the placenta, uric acid may impact amino acid transportation, inhibit trophoblast invasion, and disrupt vascular development, leading to placental insufficiency and increasing the risk of SGA. However, in the cited study, it was not significantly linked to LGA, SGA, or macrosomia.

The hemodynamic profile of GDM pregnancies impacts fetal growth. Fetal Doppler indices in GDM reflect the redistribution of blood flow consistent with macrosomia but could also reveal early adaptation patterns when growth restriction occurs. Vascular parameters, such as increased total peripheral resistance, elevated ophthalmic artery peak systolic velocity, and high uterine artery pulsatility index, were positively correlated with the development of SGA. These findings were consistent across both GDM and non-GDM pregnancies, with SGA groups exhibiting reduced cardiac output and heightened arterial stiffness. This supports the hypothesis that impaired maternal vascular adaptation plays a critical role in fetal growth restriction, particularly in the context of GDM.

Studies that have attempted to evaluate racial and ethnic disparities in the risk of developing LGA/SGA have shown inconsistent results due to the lack of ethnic-specific growth curves. Such omissions can lead to misclassifications of fetal growth. The relationship between GDM and adverse pregnancy outcomes is strongly influenced by maternal ethnicity. While some populations, such as African American, American Indian, Hispanic, and Filipino, are more prone to fetal overgrowth or undergrowth, others (Chinese, Korean, and Indian) appear to have more favorable outcomes. These findings underscore the need for tailored management based on race and ethnic groups.

A recent systematic review and meta-analysis by Li et al. [72] published in 2020, which included over 127 million participants, examined maternal age and the risk of GDM, highlighting significant associations between advanced maternal age (≥35 years) and increased GDM risk. The study found that women with GDM had a higher risk of adverse pregnancy outcomes, including LGA infants. According to a study we have included in this review, women aged 35–44 are at an increased risk for LGA and macrosomia, with advancing maternal age amplifying fetal overgrowth risks. In addition to LGA, the 40–44 group had the highest rate of SGA, showing that advanced maternal age in GDM can be linked to overgrowth and undergrowth risks. Contrasting with the previous cohort, GDM in women over 45 was associated with a lower risk of low birth weight, and no association with SGA was observed.

Our review highlights the need for clinicians to have a nuanced and personalized perspective towards the management of GDM, applying critical lens to each patient’s unique risk profile. Rather than focusing solely on the prevention of fetal overgrowth, caretakers should recognize, anticipate, and mitigate the risk of growth restriction. Consideration of maternal characteristics, such as race and ethnicity, age, pregestational and gestational BMIs, lipid profile, dietary choices, micro- and macronutrient statuses, vascular health, as well as other metabolic parameters, can help towards personalized care. Clinicians should integrate these factors into antenatal and postnatal monitoring, with the scope of reducing short- and long-term risks for both the mother and the offspring.

Given the broad scope and potential for bias inherent in narrative reviews, our findings may have limited applicability for clinical practice. GDM-affected pregnancies represent a highly heterogenous group, shaped by variations in maternal age, ethnicity, metabolic status, dietary patterns, and access to healthcare. This highlights the need for future research to prioritize not just large-scale, longitudinal studies that examine the interplay between maternal status and fetal growth patterns in GDM pregnancies but also subgroup-specific risk profiles. These studies should aim to establish causal relationships and identify critical windows during pregnancy when supplementation may be most beneficial or potentially harmful. Investigating ethnic-specific nutritional interventions is also crucial, as differences in GDM outcomes among different racial and ethnic groups suggest the need for tailored approaches, as opposed to “one-size-fits-all” management. Incorporating maternal and fetal hemodynamic profiling into routine GDM care could further elucidate vascular adaptations and their influence on fetal growth, potentially informing individualized management strategies. Lastly, standardizing diagnostic criteria for GDM and outcome measures for fetal growth would increase the comparability of studies and strengthen the evidence base for clinical guidelines.

## 5. Conclusions

Gestational diabetes mellitus is influenced by maternal metabolic, nutritional, and demographic factors. Rising obesity rates among women of reproductive age have increased the risk of GDM, posing a significant public health concern. While large-for-gestational-age infants are a well-discussed outcome of gestational diabetes, small-for-gestational-age infants also warrant attention due to their associated risks. Personalized nutritional interventions tailored to pre-pregnancy BMI, gestational timing, and metabolic profiles offer effective strategies to improve maternal and neonatal outcomes. Management should include monitoring of lipid metabolism, micronutrient status, and vascular health. Ethnicity, maternal age, and geographic context further influence outcomes, highlighting the need for culturally tailored care and public health strategies. Maternal lipid profiling shows promise for assessing the risk of fetal growth abnormalities, though further research is needed to clarify thresholds and mechanisms.

## Figures and Tables

**Table 1:** Studies identified by the comprehensive literature search via PubMed, Web of Science, and ScienceDirect.

	PubMed	Web of Science	ScienceDirect
Gestational diabetes mellitus AND small for gestational age/low birth weight/intrauterine growth restriction	295	227	1249
Gestational diabetes mellitus AND large for gestational age/macrosomia	781	142	733
Total	1076	369	1982
	3427		

**Table 2:** Main results of the included studies.

	Author, Year	Population	Outcome	SGA, Intrauterine Growth Restriction, Low Birth Weight	LGA, Macrosomia
1	Venkatesh 2022 [11]	1,560,822	race/ethnicity	The rate of SGA significantly increased for Asian/Pacific Islander and Hispanic individuals. Black individuals were also associated a higher risk of SGA.	The rate of LGA significantly decreased for all subgroups. American Indian individuals had a higher risk of LGA and macrosomia.
2	Filardi 2022 [12]	399	ethnicity	SGA was noted in 14.3% of the high-risk group (Asian, African, and Hispanic), vs. 4.8% of the low-risk population (Caucasian).	LGA was noted in 21.4% of the high-risk population, vs. 7.2% of the low-risk population.
3	Xiang 2015 [13]	29,544	race/ethnicity	The SGA risks were 11.3% in the Filipino population, 10.4% in the Asian Indian population, 9.5% in the NHW population, 9.4% in the Vietnamese population, 8.9% in the Hispanic population, 7.5% in the NHB population, and 5.6% in the PI population. Chinese, Korean, and Japanese women registered the lowest risks for SGA. After adjustments, none of the other racial/ethnic groups had a statistically significant risk of SGA compared to NHW.	The highest LGA risk was reported in the NHB population (17.2%), followed by PI (16.2%), Hispanic (14.5%), NHW (13.1%), Asian Indian (12.8%), and Filipino (11.6%). Chinese, Korean, and Japanese women registered the lowest risks for LGA. After adjustments, only NHB had a significantly increased risk of LGA compared to NHW.
4	Nielsen 2021 [14]	710,413	ethnicity/migration	Among Danish women, SGA proved to have lower odds. Forty to fifty percent reduced odds of SGA were noted in populations from countries such as the Former Yugoslavia, Turkey, Somalia, Afghanistan, Sri Lanka, Pakistan, and other non-Western countries.	The presence of GDM doubled the odds of LGA among women from all countries; this was not statistically significant among women from India or China. The odds for LGA were significantly lower among women from India, Lebanon, Pakistan, Iraq, Turkey, and Somalia compared to Danish women.
5	Zhang 2014 [15]	24,551	maternal age	The lowest rate of SGA was recorded in the 30–34 group (15.4%), and the highest rate was noted in the 40–44 group, 19.7%.	The 20–24 group had the lowest rate of LGA (14.9%), while the 35–39 and 40–44 groups had the highest rates: 21.1% and 21.2%, respectively.
6	Lu 2023 [16]	52,544	maternal age	No associations were found between GDM and SGA in women over 45 years old. GDM was associated with a significantly reduced risk of low birth weight.	N/A
7	Krstevska 2016 [17]	243	lipid parameters	DM2 group had a statistically significant higher rate of SGA (20%) compared to GDM group (7.5%), likely due to the higher percentage of preterm delivery.	Linear multiple regression analysis demonstrated that triglycerides, LDL-C, and total cholesterol were independent predictors of LGA. BMI was not independent predictor for LGA.
8	Pereira 2024 [18]	659	lipid parameters	N/A	Lower levels of HDL cholesterol were noted for women with LGA infants compared to the two other groups, as well as for women with AGA infants compared to women with SGA infants. Mothers with LGA infants had statistically significant greater pre-pregnancy BMI than mothers who had given birth to AGA and SGA infants, respectively (32.1 vs. 29.8 vs. 28.7).
9	Simeonova 2014 [19]	200	lipid parameters	Maternal triglyceride levels and HbA1c in the second trimester were significantly higher in the SGA group than in the AGA group (3.8 ± 1.9 vs. 3.1 ± 1.1 mmol/L and 6.8 ± 0.8 vs. 5.5 ± 0.8%, *p* < 0.05).	Maternal triglyceride levels and HbA1c in the second trimester were higher, and HDL-C was significantly lower, in the LGA group than in the AGA group (3.8 ± 1.8 vs. 3.1 ± 1.1 mmol/L, 6.1 ± 1.1 vs. 5.5 ± 0.8%, and 1.3 ± 0.4 vs. 1.6 ± 0.4 mmol/L, *p* < 0.05). Maternal triglycerides were independent predictors for delivering LGA newborns in GDM women.
10	Peng 2025 [20]	10,490	lipid parameters	There was no association between maternal lipid levels in women with GDM and the risk of SGA.	Compared with women with GDM with TG levels below the 10th percentile, those with TG levels over the 90th percentile had increased risks of LGA offspring and macrosomia; this risk was stronger than that in women without GDM.
11	Drever 2023 [21]	308	pregestational BMI	Women with SGA offsprings had a significantly lower median pre-pregnancy BMI (21.5 vs. 24.4). The absolute risk of having an SGA infant in women with a low pBMI was 27.3% compared to 7.9% in those who had a normal to high BMI.	N/A
12	Yang 2023 [22]	6174	pregestational BMI	Underweight women had a high risk for low birth weight and SGA.	Compared to women with a normal pBMI, women with obesity had a higher risk for macrosomia and LGA; 4.61% and 5.02%, respectively, of the associations were mediated by GDM.
13	Kondracki 2022 [23]	3,229,783	pregestational BMI	N/A	The highest prevalence of GDM was among pregestational overweight/obesity that also had the highest rates of LGA births at term.
14	Chen Xu 2022 [24]	13,467	pregestational BMI	Pregestational overweight and obesity reduced the risk for SGA. Obesity alone was associated with a decreased risk of low-birth-weight newborns.	Obesity alone was associated with a higher risk of macrosomia and LGA.
15	Liao 2025 [25]	791	pregestational BMI	The rate of the SGA babies increased with higher pre-pregnancy BMI. Neonatal birth weight displayed a decrease as maternal pBMI increased when maternal pBMI was greater than 27.78 kg/m^2^.	The percentage of LGA babies was higher in women with overweight or obesity compared to those of normal weight. Neonatal birth weight displayed a significantly increasing trend with increasing maternal pBMI when maternal pBMI was less than 27.78 kg/m^2^.
16	Saito 2022 [26]	85,228	pregestational BMI	The highest percentage of SGA was 12% in the underweight GDM group, but the OR of SGA was slightly elevated in the overweight/obese GDM group compared with the overweight/obese non-GDM group.	The incidence of LGA positively correlated with BMI.
17	Hu 2022 [27]	17,260	pregestational BMI	Compared to the maternal normal-weight group, the underweight group had 1.64 times the risk to have SGA neonates.	The overweight and obese groups had 1.79 and 2.76 times the risks of having LGA newborns.
18	Chen 2022 [28]	1428	BMI	N/A	Compared to those without GDM, subjects with GDM were 7.55 times more likely to deliver LGA babies. Women with both pre-pregnancy and pregnancy overweightness/obesity were 3.64 times more likely to deliver LGA.
19	Song 2022 [29]	15,065	pregestational BMI	pBMI over 24 kg/m^2^ was associated with a decreased risk of SGA.	pBMI over 24 kg/m^2^ had a higher risk of LGA.
20	Bruno 2017 [30]	131	maternal diet	The incidence of SGA was not different between the two groups (control and intervention).	Women who followed a hypocaloric, low-glycemic, low-saturated fat diet and physical activity recommendations had a significantly lower rate of LGA and macrosomia.
21	Apostolopoulou 2023 [31]	90	maternal diet	Higher fat intake compared to non-SGA during period B was associated with an increased risk for SGA neonates; lower intakes of carbohydrates, fiber intake, magnesium, and copper intake during period B were significantly associated with a decreased risk for SGA neonates.	N/A
22	Siargkas 2025 [32]	117	macronutrient intake	N/A	In normal-BMI women, higher dietary fiber and vegetable protein intake before pregnancy was significantly associated with an increased risk of LGA; the risk from vegetable protein persisted in early pregnancy. During early pregnancy, a higher percentage of total carbohydrate and vegetable protein intake was linked to increased LGA; low intake of saturated fatty acids reduced the odds of LGA.
23	Xie 2024 [33]	400	chol intake	In the second trimester, GDM women with high cholesterol intake had lower risks of SGA.	In the second trimester, GDM women with high cholesterol intake had higher risks of macrosomia and LGA. In the third trimester, GDM women with high cholesterol intake had lower risks of macrosomia and LGA.
24	Messika 2022 [34]	103	nutrition, sleep	N/A	The intervention had no effect on the proportion of large-for-gestational-age newborns.
25	Cheng 2019 [35]	950	folic acid supplementation	FA supplementation for ≥3 months before pregnancy was associated with an increased risk of GDM and decreased risk of SGA birth.	In the group of FA supplementation for ≥3 months during pregnancy, GDM was associated with an increased risk of macrosomia.
26	Soubasi 2010 [36]	63	ferritin	The rate of IUGR was significantly higher in the group with neonates whose mothers had high ferritin levels.	N/A
27	Weinert 2016 [37]	184	vitamin D	Vitamin D deficiency associated higher rates of SGA. After adjustment, relative risk for SGA was 4.32.	N/A
28	Zhou 2022 [38]	19,647	vitamin E	N/A	Maternal vitamin E concentrations in the first and second trimesters were positively associated with GDM and LGA
29	Pang 2023 [39]	18,250	uric acid	Serum uric acid showed a linear correlation with SGA.	Hyperuricemia is associated with a higher incidence of LGA. No significant associations were found between UA and macrosomia
30	Kim 2022 [40]	710	postprandial free fatty acids	N/A	Levels of 2h-FFA were higher in women who delivered LGA newborns than in those who delivered non-LGA newborns. Fasting FFA was not significantly different between the two groups.
31	Chatzakis 2025 [41]	11,132	vascular assessment	Total peripheral resistance, ophthalmic artery peak systolic velocity, and uterine artery pulsatility index were positively correlated with SGA. SGA groups had the highest uterine artery pulsatility index percentiles, carotid-femoral pulse wave velocity, and ophthalmic artery peak systolic velocity ratios. Compared to AGA groups, cardiac output was lower in the SGA groups.	N/A

BMI—body mass index; FFA—free fatty acid; GDM—gestational diabetes mellitus; HDL—high-density lipoprotein; IUGR—intrauterine growth restriction; LDL—low-density lipoprotein; LGA—large for gestational age; N/A—not available; NHB—non-Hispanic Black; NHW—non-Hispanic White; OR—odds ratio; pBMI—pre-pregnancy body mass index; SGA—small for gestational age; TG—triglyceride.

## Data Availability

No new data were created or analyzed in this study.

## Abbreviations

The following abbreviations are used in this manuscript:

AGA	Appropriate For Gestational Age
BMI	Body Mass Index
DNA	Deoxyribonucleic Acid
FFA	Free Fatty Acid
GDM	Gestational Diabetes Mellitus
HDL	High-Density Lipoprotein
HR	High Risk
IADPSG	International Association Of Diabetes And Pregnancy Study Groups
IUGR	Intrauterine Growth Restriction
LDL	Low-Density Lipoprotein
LGA	Large For Gestational Age
LR	Low Risk
NHB	Non-Hispanic Black
NHW	Non-Hispanic White
NTDs	Neural Tube Defects
OGTT	Oral Glucose Tolerance Test
OR	Odds Ratio
pBMI	Pre-Pregnancy Body Mass Index
ROS	Reactive Oxygen Species
SGA	Small For Gestational Age
TG	Triglyceride
WHO	World Health Organization

## References

[1] Wang H., Li N., Chivese T., Werfalli M., Sun H., Yuen L., Hoegfeldt C.A., Powe C.E., Immanuel J., Karuranga S. (2022). IDF Diabetes Atlas: Estimation of Global and Regional Gestational Diabetes Mellitus Prevalence for 2021 by International Association of Diabetes in Pregnancy Study Group’s Criteria. Diabetes Res. Clin. Pract..

[2] Paulo M., Abdo N., Bettencourt-Silva R., Al-Rifai R. (2021). Gestational Diabetes Mellitus in Europe: A Systematic Review and Meta-Analysis of Prevalence Studies. Front. Endocrinol..

[3] Veeraswamy S., Vijayam B., Gupta V., Kapur A. (2012). Gestational diabetes: The public health relevance and approach. Diabetes Res. Clin. Pract..

[4] Staynova R., Vasileva E., Yanachkova V. (2022). Gestational diabetes mellitus: A growing economic concern. Folia Medica.

[5] Buchanan T., Xiang A., Page K. (2012). Gestational Diabetes Mellitus: Risks and Management during and after Pregnancy. Nat. Rev. Endocrinol..

[6] Chen J., Xiao H., Yang Y., Tang Y., Yang X. (2021). Demographic and Clinical Features of Small-for-Gestational-Age Infants Born to Mothers With Gestational Diabetes Mellitus. Front. Pediatr..

[7] Macfarlane C., Tsakalakos N. (1988). The extended Pedersen hypothesis. Clin. Physiol. Biochem..

[8] Schmidt M., Duncan B., Reichelt A. (2001). Gestational diabetes mellitus diagnosed with a 2-h 75-g oral glucose tolerance test and adverse pregnancy outcomes. Diabetes Care.

[9] McElwain C., Tuboly E., McCarthy F., McCarthy C. (2020). Mechanisms of Endothelial Dysfunction in Pre-eclampsia and Gestational Diabetes Mellitus: Windows Into Future Cardiometabolic Health?. Front. Endocrinol..

[10] Krishna R., Bhat B. (2018). Molecular mechanisms of intrauterine growth restriction. J. Matern. Fetal Neonatal Med..

[11] Venkatesh K., Lynch C., Powe E., Costantine M., Thung S., Gabbe S., Grobman W.A., Landon M.B. (2022). Risk of Adverse Pregnancy Outcomes Among Pregnant Individuals With Gestational Diabetes by Race and Ethnicity in the United States, 2014–2020. JAMA.

[12] Filardi T., Gentile M., Venditti V., Valente A., Bleve E., Santangelo C., Morano S. (2022). The Impact of Ethnicity on Fetal and Maternal Outcomes of Gestational Diabetes. Medicina.

[13] Xiang A., Black M., Li B., Martinez M., Sacks D., Lawrence J., Buchanan T.A., Jacobsen S.J. (2015). Racial and ethnic disparities in extremes of fetal growth after gestational diabetes mellitus. Diabetologia.

[14] Nielsen K., Andersen G., Damm P., Andersen A. (2021). Migration, Gestational Diabetes, and Adverse Pregnancy Outcomes: A Nationwide Study of Singleton Deliveries in Denmark. J. Clin. Endocrinol. Metab..

[15] Zhang T., Tian M., Zhang P., Du L., Ma X., Zhang Y., Tang Z. (2014). Risk of adverse pregnancy outcomes in pregnant women with gestational diabetes mellitus by age: A multicentric cohort study in Hebei, China. Sci. Rep..

[16] Lu L., He L., Hu J., Li J. (2023). Association between very advanced maternal age women with gestational diabetes mellitus and the risks of adverse infant outcomes: A cohort study from the NVSS 2014–2019. BMC Pregnancy Childbirth.

[17] Krstevska B., Jovanovska S., Krstevkska S., Nakova V., Serafimoski V. (2016). Maternal lipids may predict fetal growth in type 2 Diabetes Mellitus and Gestational Diabetes Mellitus pregnancies. Prilozi.

[18] Pereira A., Montero M., Souza F., Jordao M., Oliveira M., Mattar R., Dib S.A., Dualib P.M., de Almeida-Pititto B. (2024). High-Density Lipoproteins-Cholesterol (HDL-C) in Women With Gestational Diabetes (GDM): A Predictor for Large Gestational Age (LGA) Babies. Cureus.

[19] Simeonova S., Krstevska B., Velkoska-Nakova V., Hadji Lega M., Samardjiski I., Serafimoski V., Livrinova V., Todorovska I., Sima A. (2014). Effect of lipid parameters on foetal growth in gestational diabetes mellitus pregnancies. Prilozi.

[20] Peng J., Zhang L., Jin J., Miao H., Liu G., Guo Y. (2025). Impact of maternal lipid profiles on offspring birth size in late pregnancy among women with and without gestational diabetes. Lipids Health Dis..

[21] Drever H., Davidson S., Callaway L., Sekar R., De Jersey S. (2023). Factors associated with higher risk of small-for-gestational-age infants in women treated for gestational diabetes. Aust. N. Z. J. Obstet. Gynaecol..

[22] Yang J., Qian J., Qu Y., Zhan Y., Yue H., Ma H., Li X., Man D., Wu H., Huang P. (2023). Pre-pregnancy body mass index and risk of maternal or infant complications with gestational diabetes mellitus as a mediator: A multicenter, longitudinal cohort study in China. Diabetes Res. Clin. Pract..

[23] Kondracki A., Valente M., Ibrahimou B., Bursac Z. (2022). Risk of large for gestational age births at early, full and late term in relation to pre-pregnancy body mass index: Mediation by gestational diabetes status. Paediatr. Perinat. Epidemiol..

[24] Chen Xu J., Coelho A. (2022). Association between Body Mass Index and Gestational Weight Gain with Obstetric and Neonatal Complications in Pregnant Women with Gestational Diabetes. Acta Med. Port..

[25] Liao Q., Yu T., Chen J., Zheng X., Xheng L., Yan J. (2025). Relationship between maternal pre-pregnancy BMI and neonatal birth weight in pregnancies with gestational diabetes mellitus: A retrospective cohort study. Front. Med..

[26] Saito Y., Kobayashi S., Ikeda-Araki A., Ito S., Miyashita C., Kimura T., Hirata T., Tamakoshi A., Mayama M., Noshiro K. (2022). Association between pre-pregnancy body mass index and gestational weight gain and perinatal outcomes in pregnant women diagnosed with gestational diabetes mellitus: The Japan Environment and Children’s Study. J. Diabetes Investig..

[27] Hu H., Feng P., Yu Q., Zhu W., Xu H., Wu D., Wu L., Yin J., Li H. (2022). The mediating role of gestational diabetes mellitus in the associations of maternal prepregnancy body mass index with neonatal birth weight. J. Diabetes.

[28] Chen H., Wu C., Hsieh C., Kuo F., Sun C., Wang S., Chen M.-L., Wu M.-T. (2022). Relationship of maternal body weight and gestational diabetes mellitus with large-for-gestational-age babies at birth in Taiwan: The TMICS cohort. Taiwan. J. Obs. Gynecol..

[29] Song Z., Cheng Y., Li T., Fan Y., Zhang Q., Cheng H. (2022). Effects of obesity indices/GDM on the pregnancy outcomes in Chinese women: A retrospective cohort study. Front. Endocrinol..

[30] Bruno R., Petrella E., Bertarini V., Pedrielli G., Neri I., Facchinetti F. (2017). Adherence to a lifestyle programme in overweight/obese pregnant women and effect on gestational diabetes mellitus: A randomized controlled trial. Matern. Child. Nutr..

[31] Apostolopoulou A., Tranidou A., Chroni V., Tsakiridis I., Magriplis E., Dagklis T., Chourdakis M. (2023). Association of Maternal Diet with Infant Birthweight in Women with Gestational Diabetes Mellitus. Nutrients.

[32] Siargkas A., Tranidou A., Magriplis E., Tsakiridis I., Apostolopoulou A., Xenidis T., Pazaras N., Chourdakis M., Dagklis T. (2025). Impact of Maternal Macronutrient Intake on Large for Gestational Age Neonates’ Risk Among Women with Gestational Diabetes Mellitus: Results from the Greek BORN2020 Cohort. Nutrients.

[33] Xie C., Zheng Q., Jiang X., Liao Y., Gao X., Zhu Y., Li J., Liu R. (2024). Association of maternal dietary cholesterol intake during the second and third trimesters of pregnancy and blood glucose and pregnancy outcome in women with gestational diabetes mellitus: A prospective cohort study. Front. Nutr..

[34] Messika A., Toledano Y., Hadar E., Shmuel E., Tauman R., Shamir R., Froy O. (2022). Relationship among chrononutrition, sleep, and glycemic control in women with gestational diabetes mellitus: A randomized controlled trial. Am. J. Obs. Gynecol. MFM.

[35] Cheng G., Sha T., Gao X., He Q., Wu X., Tian Q., Yang F., Tang C., Wu X., Xie Q. (2019). The Associations between the Duration of Folic Acid Supplementation, Gestational Diabetes Mellitus, and Adverse Birth Outcomes based on a Birth Cohort. Int. J. Environ. Res. Public Health.

[36] Soubasi V., Petridou S., Sarafidis K., Tsantali C., Diamanti E., Buonocore G., Drossou-Agakidou V. (2010). Association of increased maternal ferritin levels with gestational diabetes and intra-uterine growth retardation. Diabetes Metab..

[37] Weinert L., Reichelt A., Schmitt L., Boff R., Oppermann M., Camargo J., Silveiro S.P., Rosenfeld C.S. (2016). Vitamin D Deficiency Increases the Risk of Adverse Neonatal Outcomes in Gestational Diabetes. PLoS ONE.

[38] Zhou Q., Jiao M., Han N., Yang W., Bao H., Ren Z. (2022). The Influence of Maternal Vitamin E Concentrations in Different Trimesters on Gestational Diabetes and Large-for-Gestational-Age: A Retrospective Study in China. Nutrients.

[39] Pang T., Zhou X., Li P., Ma H., Shen X., Wan Y., Guo X.-L., Liu Z.-P., Chen G.-D. (2023). Associations of early pregnancy serum uric acid levels with risk of gestational diabetes and birth outcomes: A retrospective cohort study. BMC Endocr. Disord..

[40] Kim S., Song Y., Kim S., Cho Y., Kim K. (2022). Postprandial Free Fatty Acids at Mid-Pregnancy Increase the Risk of Large-for-Gestational-Age Newborns in Women with Gestational Diabetes Mellitus. Diabetes Metab. J..

[41] Chatzakis C., Lausegger S., Sembrera E., Vargas S., Nicolaides K., Charakida M. (2025). Maternal vascular dysfunction in gestational diabetes is associated with birth of small neonates. Diabetes Res. Clin. Pract..

[42] Haertle L., El Hajj N., Dittrich M., Muller T., Nanda I., Lehnen H., Haaf T. (2017). Epigenetic sigrantures of gestational diabetes mellitus on cord blood methylation. Clin. Epigenetics.

[43] Timmermans S., Jaddoe V., Hofman A., Steegers-Theunissen R., Steegers E. (2009). Periconception folic acid supplementation, fetal growth and the risks of low birth weight and preterm birth: The Generation R Study. Br. J. Nutr..

[44] Bai W., Wang H., Fang R., Lin M., Qin Y. (2023). Evaluating the effect of gestational diabetes mellitus on macrosomia based on the characteristics of oral glucose tolerance test. Clin. Chim. Acta.

[45] Abdelwahab M., Frey H., Lynch C., Klebanoff M., Thung S., Costantine M., Landon M.B., Venkatesh K.K. (2023). Association between Diabetes in Pregnancy and Shoulder Dystocia by Infant Birth Weight in an Era of Cesarean Delivery for Suspected Macrosomia. Am. J. Perinatol..

[46] Kekki M., Tihtonen K., Salonen A., Koukkula T., Gissler M., Laivuori H., Huttunen T.T. (2022). Severe birth injuries in neonates and associated risk factors for injury in mothers with different types of diabetes in Finland. Int. J. Gynaecol. Obs..

[47] Al-Shwyiat R., Radwan A. (2023). Fetal anomalies in gestational diabetes mellitus and risk of fetal anomalies in relation to pre-conceptional blood sugar and glycosylated hemoglobin. J. Mother. Child..

[48] Allen V., Armson B. (2007). Teratogenicity associated with pre-existing and gestational diabetes. J. Obstet. Gynaecol. Can..

[49] Millis J. (2010). Malformations in infants of diabetic mothers. Birth Defects Res..

[50] World Health Organization (2014). Diagnostic criteria and classification of hyperglycaemia first detected in pregnancy: A World Health Organization guideline. Diabetes Res. Clin. Pract..

[51] Saeedi M., Cao Y., Fadl H., Gustafson H., Simmons D. (2021). Increasing prevalence of gestational diabetes mellitus when implementing the IADPSG criteria: A systematic review and meta-analysis. Diabetes Res. Clin. Pract..

[52] Ruze R., Liu T., Zou X., Song J., Chen Y., Xu R., Yin X., Xu Q. (2023). Obesity and type 2 diabetes mellitus: Connections in epidemiology, pathogenesis, and treatments. Front. Endocrinol..

[53] Gautam R., Lkshmi R., Sharma R., Swami S. (2024). EE285 Economic Burden of Gestational Diabetes: A Global Targeted Literature Review. Value Health.

[54] Ehlers E., Talton O., Schust D., Schulz L. (2021). Placental structural abnormalities in gestational diabetes and when they develop: A scoping review. Placenta.

[55] Afsar S., Turan G., Sonmez A., Usta C., Usta A. (2023). Fetal vascular malperfusion score is linked with developing preeclampsia in women with gestational diabetes mellitus: A retrospective cohort study. Rev. Assoc. Med. Bras.

[56] Roy B. (2025). Pathophysiological Mechanisms of Diabetes-Induced Macrovascular and Microvascular Complications: The Role of Oxidative Stress. Med. Sci..

[57] Arcot A., Walker R., Gallagher K., Goldstein J., Gernand A. (2025). Gestational diabetes mellitus and vascular malperfusion lesions in the placenta: A systematic review and meta-analysis. Int. J. Gynecol. Obs..

[58] Salameh M., Oniya O., Chamseddine R., Konje J. (2021). Maternal Obesity, Gestational Diabetes, and Fetal Macrosomia: An Incidental or a Mechanistic Relationship?. Matern. Fetal Med..

[59] Puga F., Duarte D., Silva V., Pereira M., Garrido S., Vilaverde J., Moreira M.S., Pichel F., Pinto C., Dores J. (2024). Maternal Hypertriglyceridemia in Gestational Diabetes: A New Risk Factor?. Nutrients.

[60] Barbour L., Mccurdy C., Hernandes T., Kirwan J., Catalano P., Friedman J. (2007). Cellular mechanisms for insulin resistance in normal pregnancy and gestational diabetes. Diabetes Care.

[61] Nahavandi S., Price S., Sumithran P., Ekinci E. (2019). Exploration of the shared pathophysiological mechanisms of gestational diabetes and large for gestational age offspring. World J. Diabetes.

[62] Schaefer-Graf U., Graf K., Kulbacka I., Kjos S., Dudenhausen J., Vetter K., Herrera E. (2008). Maternal Lipids as Strong Determinants of Fetal Environment and Growth in Pregnancies With Gestational Diabetes Mellitus Free. Diabetes Care.

[63] Preda A., Preda S.D., Mota M., Iliescu D.G., Zorila L.G., Comanescu A.C., Mitrea A., Clenciu D., Mota E., Vladu I.M. (2024). Dyslipidemia in Pregnancy: A Systematic Review of Molecular Alterations and Clinical Implications. Biomedicines.

[64] Wang Y., Chen Z., Zhang F. (2022). Association between maternal lipid levels during pregnancy and delivery of small for gestational age: A systematic review and meta-analysis. Front. Pediatr..

[65] Zhu Y., Zheng Q.P.Y., Jiang X., Li J., Liu R., Huang L. (2024). Association between prepregnancy body mass index or gestational weight gain and adverse pregnancy outcomes among Chinese women with gestational diabetes mellitus: A systematic review and meta-analysis. BMJ Open.

[66] Chia A., Chen L., Lai J., Wong C., Neelakantan N., Van Dam R., Chong M.F.-F. (2019). Maternal Dietary Patterns and Birth Outcomes: A Systematic Review and Meta-Analysis. Adv. Nutr..

[67] De-Regil L., Fernandez-Gaxiola A., Dowswell T., Pena-Rosas J. (2010). Effects and safety of periconceptional folate supplementation for preventing birth defects. Cochrane.

[68] Lassi Z., Salam R., Haider B., Bhutta Z. (2013). Folic acid supplementation during pregnancy for maternal health and pregnancy outcomes. Cochrane.

[69] Iqbal S., Ekmekcioglu C. (2019). Maternal and neonatal outcomes related to iron supplementation or iron status: A summary of meta-analyses. J. Matern. Fetal Neonatal Med..

[70] Lucchetta R., Lemos I., Gini A., Cavicchioli S., Forgerini M. (2022). Deficiency and Insufficiency of Vitamin D in Women of Childbearing Age: A Systematic Review and Meta-analysis. Rev. Bras. Ginecol. Obs./RBGO Gynecol. Obstet..

[71] Sharifipour F., Abedi P., Ciahkal S., Jahanfar S., Mohaghegh Z., Zahedian M. (2020). Serum vitamin E level and gestational diabetes mellitus: A systematic review and meta-analysis. J. Diabetes Metab. Disord..

[72] Li Y., Ren X., He L., Li J., Zhang S., Chen W. (2020). Maternal age and the risk of gestational diabetes mellitus: A systematic review and meta-analysis of over 120 million participants. Diabetes Res. Clin. Pract..

